# When bases clash: cultural, cognitive and practical ramifications of numeration systems used in parallel

**DOI:** 10.1098/rstb.2024.0225

**Published:** 2025-10-20

**Authors:** Andrea Bender

**Affiliations:** ^1^ Department of Psychosocial Science, University of Bergen, N-5020 Bergen, Norway

**Keywords:** numeration system, numerical base, counting unit, measurement, cognition, cultural evolution

## Abstract

Numeration systems are cognitive tools that can be used to assess both discrete and continuous magnitudes (by *counting* and *measuring*, respectively). The presence and numerical value of a base in such systems have implications on several levels: They shape the system structure in creating higher (counting) units; they affect how users cognitively represent and process numerical information; and they play a role in establishing cultural conventions for standardizing systems, setting thresholds or converting between scales. This paper investigates how each of these levels is implicated when numeration systems with diverging bases are used in parallel, from small-scale traditional contexts to daily-life situations and high-stakes domains in a globalized world. In focusing on the challenges that parallel systems were designed to tackle, as well as the challenges they cause, the paper highlights bases as both a result and a tool of cultural evolution.

This article is part of the theme issue ‘A solid base for scaling up: the structure of numeration systems’.

## 1. A confusion of units

In September 1999, NASA lost a spacecraft worth US$125 million upon arrival at its destination. The *Mars Climate Orbiter* (MCO) was meant to investigate the planet’s atmosphere, collect weather data and serve as a communication relay for an upcoming mission. All had gone well when the spacecraft was launched the year before. During its approach manoeuvre, however, it ‘unintentionally deorbited’, presumably burning up when entering the planet’s atmosphere. What made the *Mars Climate Orbiter* disaster especially notorious was the reason for it: the MCO Mishap Investigation Board established ‘the failure to use metric units in the coding of a ground software file … used in trajectory models’ as the ‘one root cause’ for the loss of the spacecraft [1, p. 16]. In short, while NASA requested and expected data to be reported according to the *metric system*, one piece of software produced by a subcontractor delivered the data in *US customary units*. As a consequence, the navigation software algorithm underestimated the effect on trajectory corrections by a factor of 4.45, thus bringing the orbiter way too deep into the planet’s atmosphere ([1]; and see [2]).

This incident is not the only example of what can happen when measurement systems are misaligned. In fact, it is not even the most serious. Another prominent example is Air Canada flight 143, which ran out of fuel midair in 1983 owing to a failure to convert imperial to metric units when calculating the amount of fuel required for the journey. Remarkably, the crew managed to glide the plane to an emergency landing in Gimli, Manitoba, bringing all passengers safely to the ground, hence its nickname ‘Gimli Glider’. Way back, in 1628, owing to discrepant measurements of feet, the Swedish battleship *Vasa—*the most powerfully armed of its time—capsized without a single enemy encounter on its maiden journey, leaving 30 people dead in its wake. And let us not forget a certain navigator, who, owing to miscalculations, believed from the 1492 landfall in the Caribbean until his death that he had arrived in the East Indies.

What these mistakes and disasters have in common is that they stem from a confusion of units caused by the parallel usage of measurement systems: metric versus US customary or imperial units in the case of the *Mars Climate Orbiter* [1] and the Gimli Glider [3]; rulers calibrated to the Swedish versus the Amsterdam foot used by the different groups of craftsmen building the *Vasa* [4]; and mistaking the Arabic mile for the much shorter Roman mile when estimating the length of the Equator in the case of Columbus [5].

Now, all of the incidents listed here involve *measurement* systems rather than *numeration* systems, and *units* instead of *bases*. So why and how are they relevant for this theme issue on the structure of numeration systems? The argument to be developed here is that counting is actually one type of measuring, that systems for counting and systems for measuring intersect in interesting ways, and that a closer look at counting and numerical bases in the wider context of measuring can therefore be informative. This reasoning also allows a more comprehensive perspective on systems with diverging bases used in parallel, as well as on their cultural context, cognitive implications and practical consequences.

The remainder of this paper begins with a brief account of how counting relates to measuring, and how counting units and bases are critical properties of both types of system^1^ (§2). The paper then illustrates the role of bases for structuring systems of measurement (§3), the persistence of a non-metric system in an otherwise metric world (§4), and potential advantages that arise from introducing diverging bases (§5). The paper concludes with a brief discussion of why and how such systems come to be used in parallel and may endure despite the drawbacks (§6).

## 2. Counting and measuring

Numeration systems are cognitive tools for precise quantification, which can be applied to assess both discrete and continuous magnitudes. When assessing the cardinality of a collection of discrete items, we call the process ‘counting’; when assessing extension along a continuous dimension, such as length, mass or time, we call it ‘measuring’.

The two types of quantification differ not only according to what is quantified but also through the fact that measuring an extension typically requires a material tool (e.g. a ruler for length, scales for mass, a clock for time) whereas counting does not. Nevertheless, the two are interchangeable to a certain extent. For instance, counting might not always be the best strategy for quantifying discrete items: if you were to ask me whether my brother or I picked more lingonberries, neither of us would bother counting them; instead, we would opt for a measurement involving volume or weight. Conversely, counting might complement measuring [6, p. 715], such as when adding together the number of units of a continuous magnitude in which something was measured (say, mugs of water), rather than measuring the magnitude in total.

Despite their differences, the two types of quantification also share essential features. Both counting and measuring turn the perception of an imprecise magnitude (say, ‘many lingonberries’, ‘a long boat’) into a *precise* representation (‘25 lingonberries’, ‘a 25 m boat’). In order to establish the numerical value of the magnitude, either type of quantification requires a *reference unit* and a *scale* along which the number of units can be assessed, which can both vary with context. Finally, the numerical *values* achieved in either type are imported from the underlying numeration system.

For the purpose of illustration, let us consider a pile of shoes. To quantify the shoes, we first need to settle the *unit* question: are we interested in the number of individual shoes, pairs of shoes, or types of shoes? We can then map, say, the pairs of shoes onto the *scale* with a one-to-one correspondence, that is, the conventionalized counting sequence in our preferred language: ‘one, two, …, thirteen pairs of shoes’. If we were interested in how long the line of shoes is (one placed after the other), we would not trouble ourselves with pairs or types. Instead, we would use a tape measure, with its predefined units, to map the length of the line of shoes and read off the number of units: ‘702 cm’. And because units for both counting and measurement are culturally defined and variable, the outcome might alternatively be ‘26 individual shoes’ or ‘276 inches’, respectively.

In theory, numeration systems are independent of whatever is to be quantified and neutral to the task. However, they were, at some point, developed for a purpose and have been shaped by cultural evolution. Arguably, the primary use for which the first systems were invented was to count discrete items (and they were only subsequently extended to measuring continuous expansion); and in practice, counting still tends to be the task for which they are applied in their ‘purest’ form. This is reflected in how the more extensive systems became structured by marking, and recursively using, increasingly higher units, for instance in the form of a base and increasingly larger powers. And the more generalized and abstracted from its initial uses the counting system becomes, the more the mathematical structure is highlighted—hence the labelling of the numeration system implemented in English as decimal and of its higher units as powers of its base.

### (a) (Counting) units and bases

It is rather uncommon to think of numerical bases as counting units, let alone as units of measure. After all, the base of a numeration system may appear to be just one among many numerals, referring to one of many numbers greater than 1. In today’s dominant systems, that number is 10. By contrast, the primary counting unit with which most counting and measuring systems operate is almost uniformly set to 1, that is, 1 discrete item when counting (an exception is described in §5a) or 1 unit of extension when measuring (1 m, 1 g, 1 h, etc.). Note that the value of 1 for the primary counting unit applies regardless of its actual magnitude; even when counting things in scores (or pairs or bundles), the unit being counted with is still 1, as in 1 score (or pair or bundle) rather than 1 single item.

Yet, when applied to larger magnitudes, neither system can exclusively rely on its primary counting unit. It is when they introduce higher counting units in a systematic manner that the systems effectively establish a base-like component. This is also reflected in how the glossary for our special issue defines *base*:

a numeration system only has a base when the system represents a natural number *b* in a way such that (i) **powers of** *b* (**including** *b* **itself) are used as counting units** and (ii) numerals for powers of *b* have distinct representational status within the system.

(*Glossary* [7], boldface added; and see [8], for more details.)

Mathematically speaking, a base is the number which is raised to various powers: For 365, written as 3 × 10^2^ + 6 × 10^1^ + 5 × 10^0^ in polynomial form, 10 is literally the base for each power expression, and is indeed recognizable as such. This is not the case in most traditional numeration systems across representational formats. The number implemented as the base can, but need not, be specifically marked. Whereas ‘10’ written in Western numerals^2^ is marked by position ( = 1 × 10 + 0 × 1), ‘ten’ in spoken English is just another *non-*marked atomic numeral. In the case of ‘ten’, it is only its recursive use in composing numerals for numbers above 10 (and the fact that its powers too are distinctly marked) that gives it away—top-down so to speak—as the base.

Instead of explicitly marking a base and its powers as such (e.g. in the polynomial above), most counting and measurement systems simply establish higher units. Take a number expression like the Norwegian word for 60, *seksti* (literally ‘six ten’): this is typically transcribed as ‘6 × 10’ because its numerical value results from a multiplication of 6 and 10. Yet, there is no element in the numeral that explicitly indicates multiplication, either in Norwegian or in most other languages. Instead, numerals such as ‘ten’ or ‘hundred’ are enumerated themselves, as if they were discrete items:


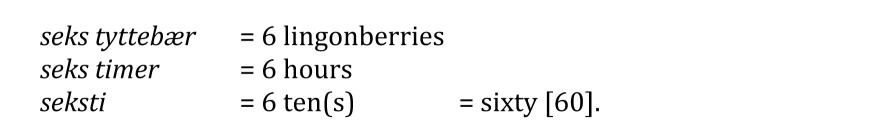


This treatment of bases and their powers as higher counting units (potentially even in a recursive manner, as detailed in [10]), is perhaps most transparent in languages that use numeral classifiers. In such languages, numerical expressions consist of two components: an atomic numeral for the numbers below the base and a numeral classifier. Things to be quantified are treated as some kind of mass, and the classifier assigns a unit to this mass [11]. To illustrate this with examples from the Micronesian language Chuukese [12]: when quantifying ‘coconut-stuff’, the speaker has to specify the unit of quantification as trees, leaves, fruits or any other dimension of ‘coconutness’:


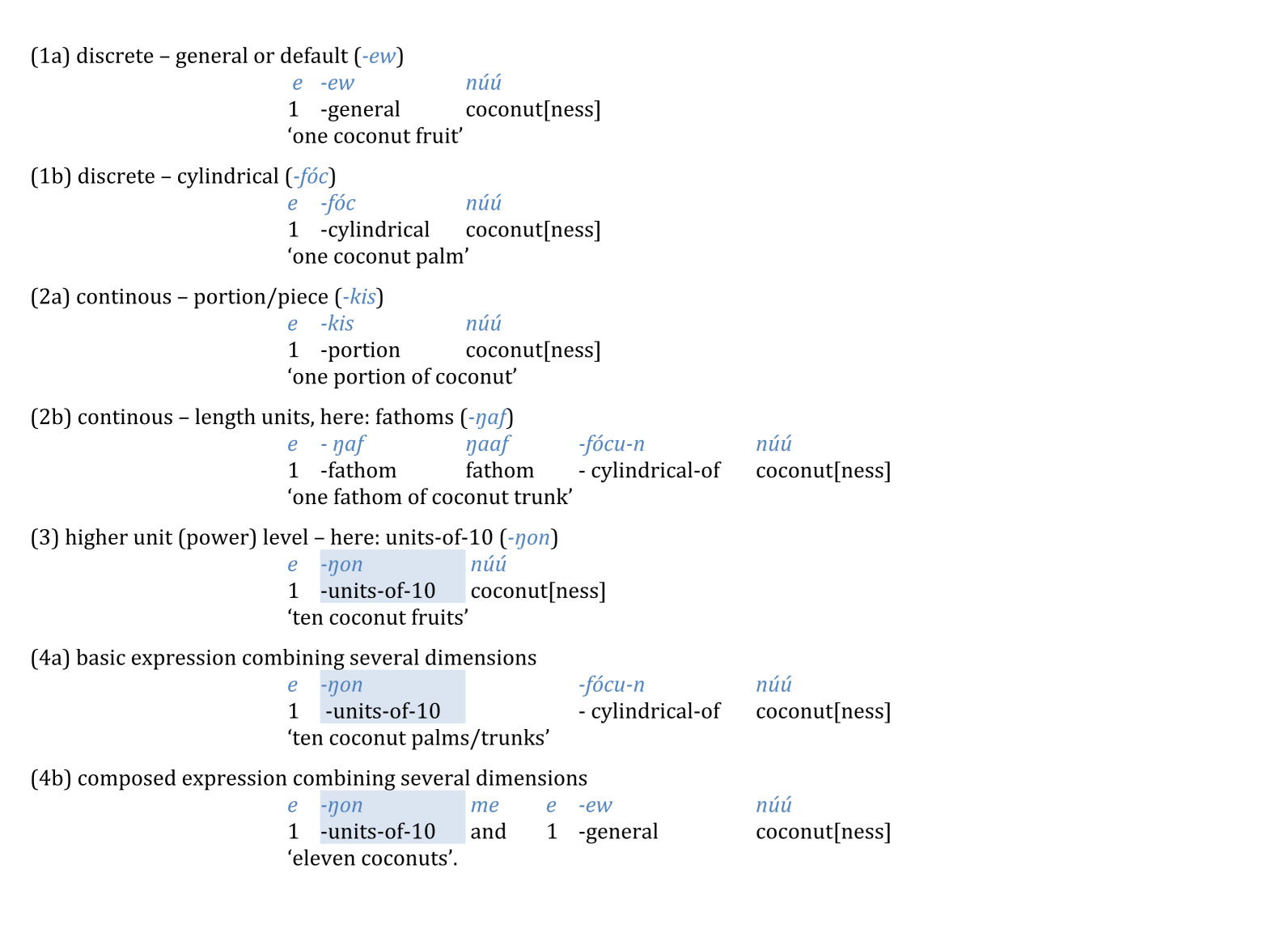


If continuous, the classifier specifies a unit of measure, as in (2a) and (2b). If discrete, the classifier typically indicates an individual item, (1a) and 1(b), but may also refer to kinds, parts, bundles or groups, or multiples such as pairs or scores, as well as—and crucially for the argument developed here—the base and its powers, as in (3) ([12]; and see [13,14]).

By implementing additional units of quantities or extensions in a systematic manner—as with ‘hundred’ and ‘thousand’, or ‘centimetre’ and ‘millimetre’—these systems behave as if they are operating with a base proper. Still, in the examples discussed here, this pattern is imported from the underlying mathematical structure of the numeration system (in §3, it will be illustrated that this need not always be the case).

### (b) Structural and cognitive implications of bases

In a numeration system, the presence of a base and its numerical value have implications on several levels, including for the mathematical structure of that system and for how its users cognitively represent and process numerical information [15,16].

Whether or not a system contains a base to begin with determines its structure: introducing a base turns a linear (one-dimensional) sequence of numerical symbols into a structured (two-dimensional) system, in which the base and its powers are re-used for composing higher numerals [16]. Such two-dimensional systems allow for a more compact representation of larger numbers than one-dimensional systems do, which also facilitates the extension of the system by orders of magnitude. At the same time, the additional dimension increases the potential for irregularities—as, for instance, in composed number expressions, where addends might come in inverse order (e.g. in English numerals ‘thirteen’ to ‘nineteen’ for 13 to 19, or German numerals up to 99, in which the units are mentioned before the tens)—which again poses considerable challenges for those learning the German numerals ([17–19]; for a review, see [20]).

Besides the mere presence of a base, its numerical value has implications too. Most obviously, this value determines which arithmetic operations are more straightforward, with addition and multiplication of that very value being easiest in every system. Other factors depend on the concrete value of the base: base-10 systems, for instance, afford simple division by two numbers (2 and 5), whereas base-12 systems afford four (2, 3, 4 and 6). This is presumably why many measurement systems—even in the presence of a decimal counting system—have operated on base 12 [6].

Base size also determines the addition and multiplication tables: ceteris paribus, the larger the base, the more elementary facts need to be learnt for arithmetic, and the greater the potential for errors (e.g. a binary system implies three elementary facts, a decimal system 55 and a vigesimal system 210). This increase in workload and learning demands, however, is offset by a more efficient encoding of numerical information. As systems with larger bases require, on average, fewer symbols carrying numerical meaning (*numemes*) to represent any number [21], their representations are more compact and hence allow more efficient processing [16,22].

### (c) Bases as benchmarks

The number used as a base to structure a numeration system not only is one of the system’s most salient numbers, but gains additional, psychologically relevant qualities. Bases and their powers are numbers to which we round up or down when precision is not essential [23], and are therefore much more frequent in discourse [24–26].

They also create categorical boundaries for otherwise continuous dimensions, such as the *p-value* used as the threshold for statistical significance in the social sciences. Arbitrarily set at 0.05 (= half of a 10th for statistically ‘significant’ results) when it was first proposed—now frequently extended to 0.001 = a 10th of a 10th of a 10th, for ‘highly significant’ results—this value has turned into a benchmark that divides the numerical continuum into two distinct ranges, overemphasizing the distance between numbers that are above and below this value [27]. Similar boundary effects are demonstrated for other base-related numbers, such as ‘thousand’ and ‘million’ [28]. Benchmarks depend on the concrete system used for measuring and can therefore differ in absolute size across cultures, such as for temperature measured in *Fahrenheit* versus *Celsius* or for speed limits measured in *mph* versus *km h^−1^*. For instance, the two default speed limits in Germany are 50 km h^−1^ (31.069 mph) within towns and 100 km h^−1^ (62.137 mph) outside built-up areas, whereas these limits in New Jersey (USA) are set to 25 mph (40.234 km h^−1^) for urban roads and 50 mph (80.467 km h^−1^) for rural roads.

In short, bases are central to people’s perception of quantities, their notions of numbers, and how they cognitively represent and process numerical information, including the everyday mathematics in which they engage. It is precisely this central role that leads to challenges when having to deal with *diverging* bases across systems. Most obviously, calculation across such systems requires conversion of numerical values, as the value of each composed numeral is based on constituents with diverging values. For instance, to grasp the numerical value of 2024 in our familiar base-10 system, we need to compute 2 × 1000 + 2 × 10 + 4 × 1, whereas its equivalent in a base-20 system requires us to compute 514_20_ = 5 × 400 + 1 × 20 + 4 × 1. Learning to decompose, let alone ‘see’, the numerical value of a numeral based differently is difficult [20]. Furthermore, benchmark values ‘mean’ more in the system with which one is more familiar (as was driven home when the members of the Euro Zone adopted a shared currency and consumers became confused by the new prices of their everyday grocery shopping).

In severe cases, like the ones listed in the Introduction, the coexistence of diverging systems can therefore incur substantial (and occasionally fatal) costs. Some of these challenges will be elaborated in the following.

## 3. Bases in measurement systems

As argued above, the key units in measurement systems can act like bases. However, not all of them do so. Here, these types of measurement systems are contrasted in order to highlight their diverging implications.

### (a) Regular systems

A measurement system is termed *regular* here if its marked units build on one another in a systematic manner, in that (i) they derive from a reference unit and (ii) higher units are related to the preceding unit by a constant factor (its ‘base’) and, hence, to the reference unit by a power of this base.

To illustrate the principle, let us turn to the world of modern communication and computing. In this world, digital information is the key commodity, and the systems for quantifying this commodity are built on units of bits and bytes. The *bit* (short for ‘binary digit’) is the lowest possible unit, taking a value of either 0 (off) or 1 (on). The most *meaningful* basic unit, however, corresponds to the number of bits that are required to encode symbolic information in a computer, at the level of single characters in a text. Historically, this tended to be 2^3^ = 8 bits = 1 byte (or 1B), and being the smallest addressable unit of memory, it became the reference unit of the system.

How higher units are defined depends on which protocol is adopted. Some systems follow the decimal pattern recommended by the *Système International d'Unités* (SI) in recruiting 10 as the base from the *byte* upward and using the decimal SI prefixes such as kilo-, mega- and giga- (hence 1 *kilobyte* (1 kB) = 1000 (10^3^) *bytes*). Others retain base-2 throughout, and most of these also specify the prefixes as binary, as in *kilobinary-* (hence 1 *kibibyte* = 1024 (2^10^) *bytes*). The following conventional, yet non-canonical, system combines the binary pattern with the decimal SI prefixes in an idiosyncratic manner:


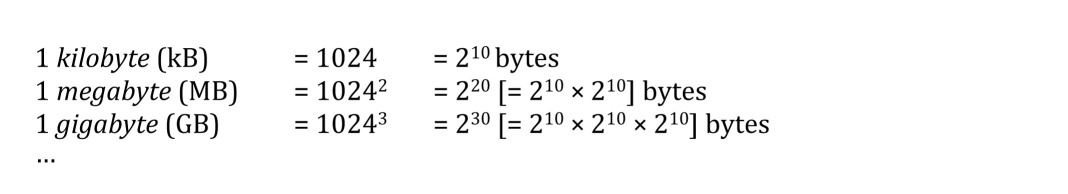


While the least consistent, this system is nevertheless the most widely known, as it is used, for instance, in various telecommunication services and in the operating system of Microsoft Windows.

The coexistence of these diverging systems for quantifying digital information can be confusing and has practical consequences. When consumers buy IT equipment, a major concern is computer storage capacity, typically rendered in units such as MB, GB, etc. However, the decimal versus conventional reading of these units produces different numerical values, and this difference increases further with size. Finding themselves mistaken about what the units refer to has led consumers to become sufficiently frustrated to file lawsuits in the USA (https://en.wikipedia.org/wiki/Byte, accessed 10 Oct 2024).

One reason for choosing the conventional system as an example above is to point out that *regular* need not, in principle, imply *decimal,* but might in fact recruit *any* base size. Still, the most regular systems in place today around the globe—the metric units regulated in the SI (for a section, see table 1)—happen to be decimal as well. This choice of base size was not a natural necessity, but neither was it coincidental. The conversion to the metric system (*metrication*), was grounded in base-10 simply because it affords straightforward computations of measurement values with the numeration system already in place for counting and calculating.

**Table 1.** A section of the Système International d'Unités (SI), illustrated for one of its seven fundamental quantities: length (‘…’ indicates regular continuation).

numerical value		SI convention		example	
power level	factor	prefix	symbol	length	English numeral
…	…	[…]		…	
10^9^	1 000 000 000	giga-	G	gigametre	billion/milliard
10^8^	100 000 000	*—*		*—*	(hundred million)
10^7^	10 000 000	*—*		*—*	(ten million)
10^6^	1 000 000	mega-	M	megametre	million
10^5^	100 000	*—*		*—*	(hundred thousand)
10^4^	10 000	*—*		*—*	(ten thousand)
10^3^	1 000	kilo-	k	kilometre	thousand
10^2^	100	hecto-	h	hectometre	hundred
10^1^	10	deca-	da	decametre	ten
**10** ^**0**^	**1**	—	—	**[metre]**	**[one]**
10^−1^	0.1	deci-	d	decimetre	tenth
10^−2^	0.01	centi-	c	centimetre	hundredth
10^−3^	0.001	milli-	m	millimetre	thousandth
10^−4^	0.000 1	*—*		*—*	
10^−5^	0.000 01	*—*		*—*	
10^−6^	0.000 001	micro-	μ	micrometre	millionth
10^−7^	0.000 000 1	*—*		*—*	
10^−8^	0.000 000 01	*—*		*—*	
10^−9^	0.000 000 001	nano-	n	nanometre	(part in a billion)
…	…	[…]		…	

This tight link is also reflected in the fact that the levels at which a new prefix is introduced parallel those at which a new power numeral is introduced in English: just as the verbal system lacks power numerals for 10 000 and 100 000 (and any other higher power level that is not a multiple of 3), so too does the SI not specify prefixes for these same ranges. As a consequence, both types of systems jump in steps of 1000 (instead of 10) beyond ‘thousand’ and *kilo-* (which, more precisely, renders both of them instances of a base-1000/sub-base-10 system).

Not all measurement systems have larger units distinctly labelled, such as the systems for measuring temperature in either degrees Celsius (°C) or degrees Fahrenheit (°F). Those lack the base-like structure discussed above. Take the following statement from a NASA website: ‘The hottest part of the Sun is its core, where temperatures top 27 million °F (15 million °C)’ (https://science.nasa.gov/sun/facts/). Of course, ‘the numbers’ still follow a base-10 pattern with regard to the numerical value expressed (as ‘27 million’ or ‘15 million’, respectively), but this does not apply to the unit of measure: speaking of, say, ‘27 mega-Fahrenheit’ would sound pretty odd. Adopting the SI-defined metric prefixes, the Kelvin scale, by contrast, permits phrasings as the following: ‘The coronal plasma is roughly around 1 mega-Kelvin (MK) but can reach temperatures of around 10 MK in certain regions’ [29].

### (b) Non-regular ‘systems’

**Figure 1.**
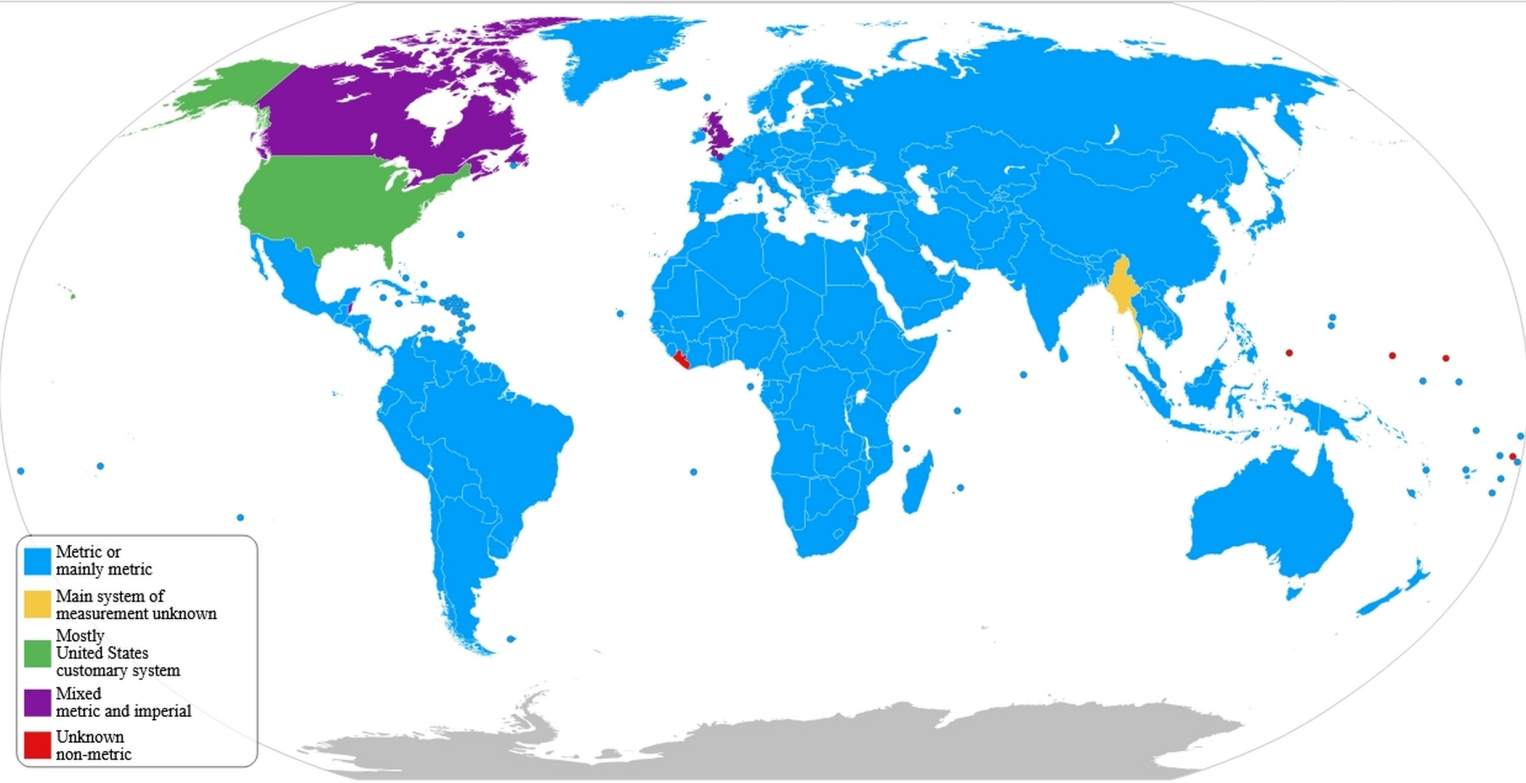
Countries using the metric (SI), imperial and US customary systems as of 2019 (by Goran tek-en, CC BY-SA 4.0, https://commons.wikimedia.org/w/index.php?curid=96077271).

Today, the (decimal) metric system of measurement following the SI is the internationally accepted standard, with very few exceptions (depicted in figure 1). Getting there was not easy though. For most of human history, hybrid collections of smaller ‘systems’ were used instead, which were not (easily) convertible, either internally across units or externally into one another [6,30]; and even many of those qualifying as systems preferred alternative bases, including 12, 16, 20 and 60 [31]. Despite the verbal systems in most European languages being largely decimal, and even after the introduction of a fully decimal notational system, it took centuries to eventually enforce standards for decimal systems of measurement (for historical overviews, see, e.g. [32–35]; for a cognitive perspective, see [36]).

The most prominent conglomeration of non-metric systems still in use is to be found in some major English-speaking countries. The length measures listed in table 2, which are used in both the *Imperial System of Units* and the *US Customary Units*, are grounded not in units derived systematically from one reference unit, but rather in distinct sets of units, each specified in its own terms. And while, over time, most of these units came to be defined such that they can be converted into one another, the numerical operations involved differ across almost all steps (e.g. 4 inches render 1 hand, 3 feet 1 yard, 22 yards 1 chain; for an abacus representation reflecting the more regular parts of some of these systems, see [37]).

**Table 2.** Major units for measuring length as in use in the UK, Canada and the US, set in relation to various reference units, including the metre in the metric system.

unit			relation to …			
name	abbreviation	symbol	previous	other reference unit	foot	metre
twip				^1^/_1440_ in	^1^/_17 280_	0.0000176389
thou/mil	th/mil		1.44 twip	^1^/_1000_ in	^1^/_12 000_	0.0000254
barleycorn	Bc		333^1^/_3_ th	^1^/_3_ in	^1^/_36_	0.0084667
inch	in	''	3 Bc	1000 thou	^1^/_12_	0.0254
hand	h, hh		4 in		^1^/_3_	0.1016
**foot**	**ft**	*'*	3 h	12 in	**1**	0.3048
yard	yd		3 ft		3	0.9144
chain	ch		22 yd	^1^/_10_ fur	66	20.1168
furlong	fur		10 ch	220 yd	660	201.1680
mile	mi		8 fur	5280 ft or 1760 yd	5280	1609.3440
league	lea		3 mi	15 840 ft or 5280 yd	15 840	4828.0300

Having standardized metric units does not prevent the continued usage of non-metric units: in the UK and Canada, imperial units are still commonly used in daily life, alongside the officially adopted metric system. And in the US, customary units dominate in commercial activities and personal use, whereas usage of the metric system is restricted to science and to some administrative and military sectors. In some situations, there may be good reasons for preferring non-metric units over their metric counterparts, especially where ergonomic concerns loom large [30], and powerful mechanisms can keep such practices in place [6,9].

But this may also come at a cost, and it is when things *do not* work that we get a clearer idea of *how* they work. The incidents reported in the Introduction attest to such costly usage of diverging measurement systems: metric versus US customary and imperial (in the case of the *Mars Climate Orbiter* and the Gimli Glider) and a different calibration of non-metric units of length (when building the *Vasa* and in Columbus’s computations of Equator length). Common to these four cases is that the fatal confusion was brought about by conflicting cultural traditions. It is precisely in order to solve such issues that international standards were established.

## 4. Non-metric measuring in a metric world: the oddity of time-reckoning

The single exception to the metric units in the SI are those for measuring time. Although time contains the second (s) as the reference unit and allows for the representation of larger units by powers of that unit (e.g. 1000 s as 1 *kilosecond* (ks)), the traditional units listed below in blue are still widely accepted:


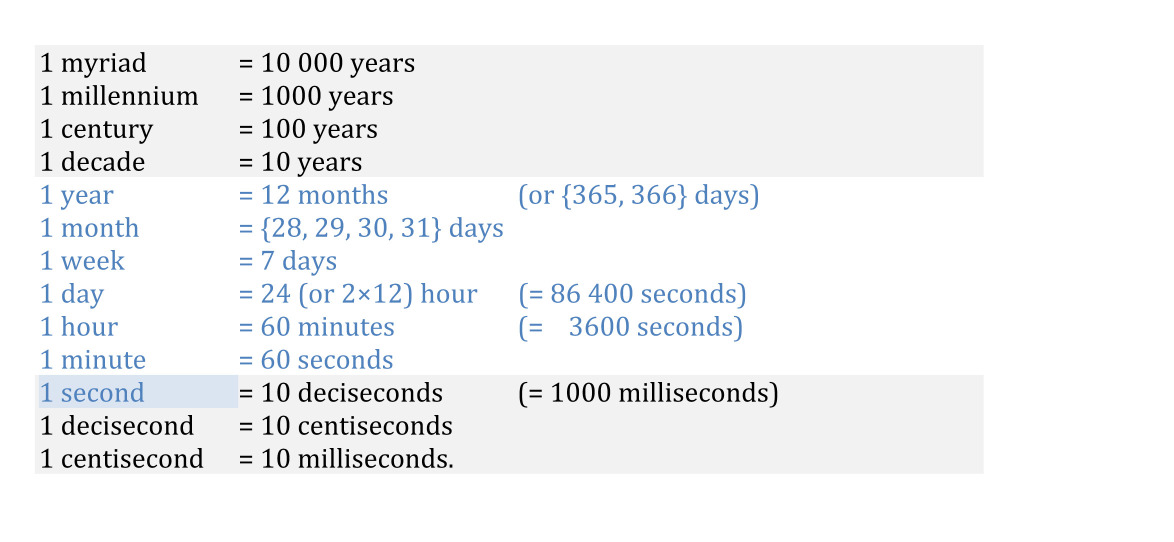


The core of this system, ranging from the second to the year, is neither regular nor metric, as its units are not systematically related (not even at a fixed ratio in the case of days in a month), and not a single step in the core sequence is based on 10. Only above the year and below the second—that is, at scales not normally relevant in daily life—do units become decimally extended.

The core system accounts for the non-decimal rhythms of solar and lunar cycles, seasons and days, while also recruiting culturally revered numbers where possible. And as some of its units are linked to the planet’s rotation and the ensuing alteration of daylight, time measurement is also relative to geographical location. By adjusting to such external, natural parameters, the hours of a day end up being spread over diverging time zones around the globe. This was hardly a major issue in the past, but grew into one with the advent of telecommunication and fast long-distance travel.

In addition to such large-scale implications, the non-metric measuring of time also has lower-level practical and cognitive implications. Perhaps the most trivial is that distinct rules for notation are recommended to avoid confusion: a colon instead of the comma (or point in most English-speaking countries) to indicate smaller units of clock time (as in 11:45), and primes for smaller units of time intervals (as in 1 h 20′ 35″). More importantly, it also complicates conversions and computations across units within time. For illustration, let us add the flight times of 1 h 50 min and 2 h 50 min, the result of which is *not* 1.50 + 2.50 = 4.00 (as in 4 h). Instead, this calculation requires, first, correct notation and then a transformational step: 1:50 + 2:50 (= 3:100) = 4:40 (4 h 40 min). Such issues also encumber conversion in derived systems, including those for measuring velocity or acceleration: to roughly which speed limit in km h^−1^*—*or indeed mph for that matter—does a wind force of 8 m s^−1^ correspond?

Today’s absence of decimal time in the SI does not imply that nobody was able to envision it. Carrigan [38] and Vera [31] trace the history of decimal time in Europe from incipient ideas to actual attempts to introduce it, most notably during the French Revolution. In 1793—or ‘Year II of the Republic’ according to the French Republican Calendar system [39]—a new time-reckoning based on the following units was put into effect:


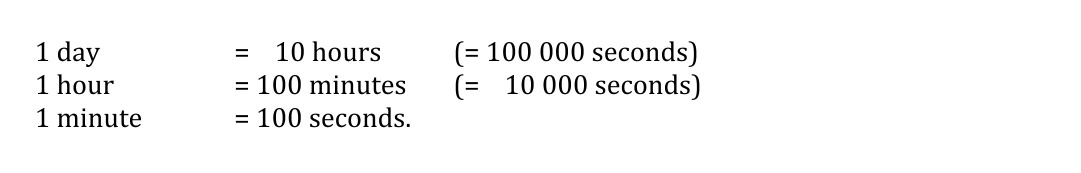


Yet, despite being clearer, simpler and more straightforward, decimal time-reckoning never caught on in administration and among the population more generally, either during its brief initial existence (decimal time-reckoning was abandoned after just 17 months, the new calendar after little more than a decade) or upon later attempts to establish it [31,38]. Although the French continued to push for it, including at the International Meridian Conference in 1884 and during every single step toward standardization, not even scientists and engineers seemed to warm to it. This is all the more noteworthy given that all of the projects with which it was coupled—the metrication of weights and measures, decimal currencies, even the standardization of time zones—*were* successful [31].

Among the few domains in which decimal measurement of time has actually managed to gain a foothold are those in need of independence of geographical location. In aviation, time is tracked by recording decimal fractions of hours, and in astronomy, observations in space are recorded in fractional days [38]. A third, much more down-to-earth domain, is populated by companies billing on an hourly basis, where even small individual miscalculations swiftly mount up to substantial expenses—so much so that computational aids are now on sale for tackling this problem.

## 5. Reshuffling bases within and across systems

The parallel usage of systems with diverging bases is mostly noticed and discussed when it causes problems, and following this tradition, the first part of this paper analysed some of the challenges that give rise to such problems. However, the same challenges can also provide opportunities and bestow practical benefits, as suggested by the continued coexistence of different systems around the world. Indeed, the Pacific is brimming with island societies that did not just let diverging systems persist but even deliberately created them. This section showcases how bases can be, and have been, reshuffled to address specific needs.

### (a) Specific counting systems in Mangarevan

Particularly in Polynesia and Micronesia, two types of verbal systems were used in parallel: a regular and fully decimal system for counting in general and one or more systems restricted to counting a few objects in a distinct manner [13,40–42]. These *specific counting systems* were all derived from the general one, but they employed primary counting units with a magnitude greater than 1, and some of them operated with bases other than 10.

**Figure 2.**
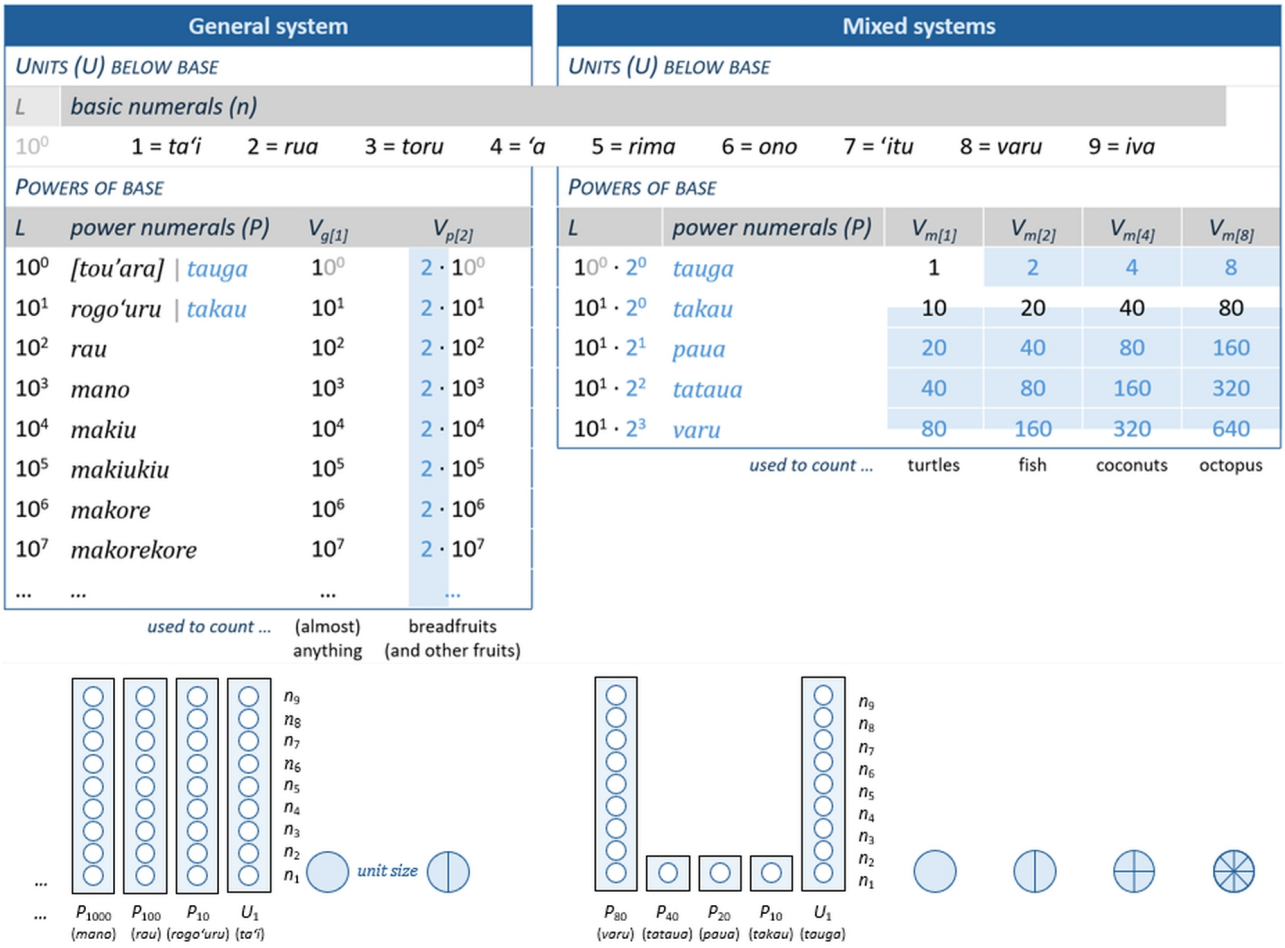
The traditional numeration systems in Mangarevan (adapted from [21]). *Top:* Key elements of the verbal systems (*L* = power level, *V* = total numerical value of the collection, with subscripts *g*, *p* and *m* indicating the general, pair and mixed systems, respectively, and numbers in square brackets indicating the size of the counting unit; binary elements in blue). *Bottom:* Visualization of base structure with the abacus representation proposed by Schlimm [37].

For illustration, take the Polynesian language spoken on Mangareva, an island group in the southern Pacific [21,43,44]. In pre-colonial times, Mangarevan contained a general system, used either for ordinary or pair counting, and four mixed systems. All of them share the basic numerals for 1 through 9, but they deviate from one another and from the general system in several ways: in the magnitude of their primary counting unit (called *tauga* in all but the general system), in the number of and numerals for their higher units, in the numerical value and interrelations of those units, and in the objects to which each system applied (figure 2).

While the *general system* operates on units of 1, when used in pair counting its numerals refer to units of 2. Any composed number word *N* was construed by adding terms on different power levels as follows:


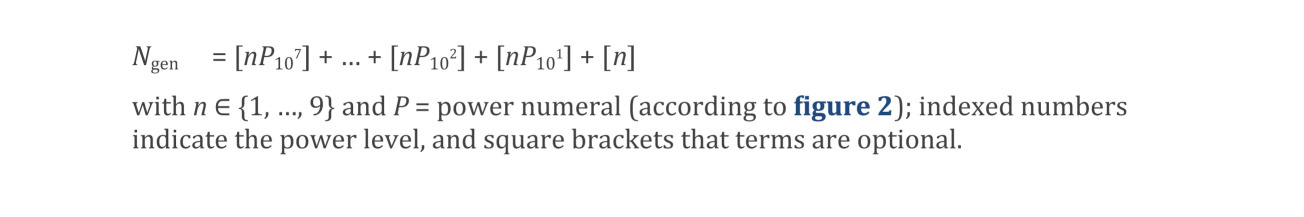


For instance,


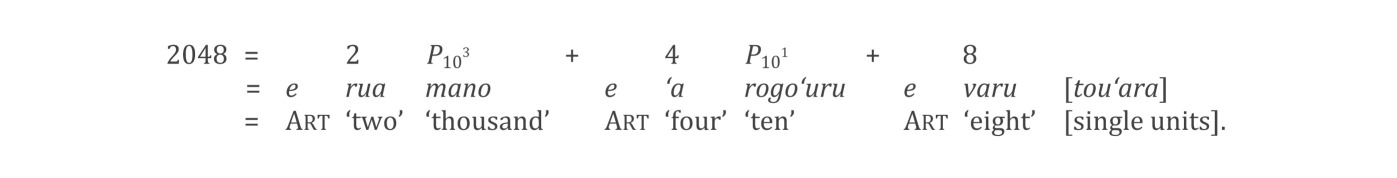


When applied for pair counting, the only element that changes is that [*n*] gets extended to [*nU*_1_], with *U*_1_ = primary unit *tauga*.

The *mixed systems*, too, differ from one another in the size of the collection counted (consisting of 1, 2, 4 or 8 individual items). More importantly, they also differ from the general system by dissecting the range between 10 and 80 into binary steps.

Any number word *N* in these systems was thus composed as follows:


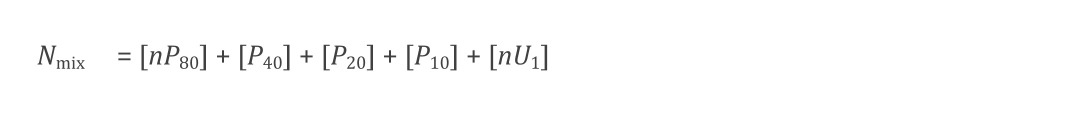


The same number as above, yet achieved when counting coconuts, would thus be


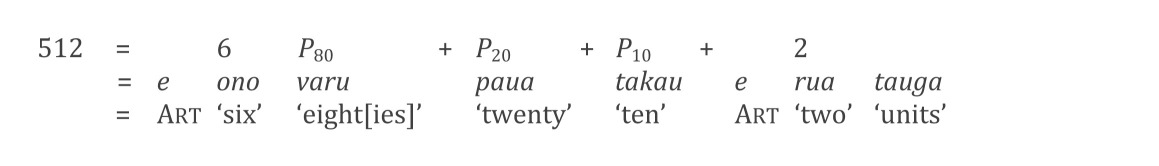


and as coconuts are counted in units of 4, this would correspond to a total of 2048 coconuts.

In all of these systems, thus, the numerical expression *N* indicated the number of the counting units, yet in some of them, obtaining the total value required further multiplication with unit size.

### (b) Implications of hybrid system structure

It is unknown how these multiple systems were employed, as they ceased to be in use at the beginning of the last century. Still, a representational analysis in the spirit of Zhang & Norman [16] (and see [45–47]) reveals their cognitive implications, and what might appear cumbersome at first glance turns out to be cognitively advantageous, with larger counting units and binary steps contributing to these advantages in a complementary manner.

#### (i) Implications of larger counting units

The general system, when recruited for pair counting, and three of the four mixed systems set their primary counting units to powers of 2: pairs (2^1^), quadruples (2^2^) and octuples (2^3^). Increasing unit magnitude in this way extracts factor 2, 4 or 8, respectively, from the numerical expression: 24 breadfruits become 12 (pairs of) breadfruits, 24 coconuts 6 (quadruples), and 24 octopuses 3 [octuples]. In other words, the representation of quantity gets divided between two distinct components: one part *explicitly* in the numeral expression itself, and the other *implicitly* in the counting unit. This renders the explicit representation more compact on average, as it comprises fewer symbols carrying numerical meaning (*numemes*) to be represented and processed [21]. For the 24 coconuts above, this would boil down to just one numeme (*ono*_[=6]_) instead of three (*rua*_[=2]_ *rogo’uru*_[=10]_ *‘a*_[=4]_). In the absence of notational systems, as in pre-colonial Mangareva, such a reduction of numemes helps lessen cognitive load [21,44], which in turn increases the speed and correctness of mental arithmetic [22].

This extraction corresponds to how numerals for the base and its powers are implemented, while carrying it one step further: just as numerals like *hundred* or *rau* constitute higher units (consisting of a certain quantity of items) to be counted themselves, so too can the primary unit of the counting (here: *tauga*) consist of a certain quantity of items. The distribution of numerical value, not just to base and power, but also the primary unit, extends the specific counting systems of Oceania beyond the two standard dimensions described earlier. In introducing unit size as an additional dimension, these systems effectively tackle the trade-off for base size: by allowing users to cognitively represent and process increasing quantities without increasing cognitive load.

#### (ii) Implications of binary steps

Whereas the general system—even when recruited for pair counting—retains its purely decimal structure, the four mixed systems inserted three binary steps. Again, we lack concrete ethnographic evidence as to how these systems were put to use, but it is undeniable that binary relations facilitate basic operations, as already demonstrated by Leibniz [48].

For instance, addition in a binary system gets by with two operations: transformation to a higher power level when an equal number of constituents is added, and/or concatenation of constituents otherwise [48]. Along similar lines, systems based on powers of 2 are exceptionally suitable for simple operations such as multiplication (e.g. by recurrent doubling) and division (by recurrent halving) [6,49,50]. How such operations would have worked out when computing numbers in the Mangarevan systems, and how this relieves working memory, are described in detail by Bender & Beller, in [44] and [21] respectively. This advantage even persists when the binary steps are integrated into an otherwise decimal system [44].

Why would a small-scale traditional society like Mangareva provide a context in which such refinements thrive? Well, Mangareva was one of the most highly stratified societies in Polynesia, where goods were accumulated in substantial quantities during tribute-giving, redistribution and trading [44]. Keeping track of and calculating large quantities in the absence of notation is cognitively taxing. The mixed systems, with their combination of decimal and binary steps, were remarkably well placed to tackle these challenges, as the joint benefits of the two bases offset their downsides: while the decimal parts keep representation compact, hence relieving cognitive load in retaining information, the binary parts reduce the number of addition facts, hence relieving cognitive load in processing.

Mixed-base systems involving binary sequences are not uncommon. An ancient Chinese system for measuring length, for instance, combined binary steps in the centre of the system—‘the space of convenient quantities’—with decimal sequences towards the lower and upper end [6, p. 720]; and in Mali, the verbal numeration system of Supyire combines two binary sequences with sequences anchored in 4 and 5 [37]. In fact, similar patterns are so recurrent in customary measurement systems around the world, particularly in those where direct one-to-one comparison is easy to establish, that Whitman [6] considers binary relations their major organizing principle. What may still make Mangarevan unique is that it instantiates the principle twice: in counting units set to powers of 2 and in the relation between some of the higher units in the mixed systems.

## 6. Discussion

Numeration systems with diverging properties have coexisted in the same cultural context throughout major parts of human numerate history. They still do so today. In Western Europe, for instance, this involves multiple systems across representational formats, notably spoken number words, Western numerals, a 10-finger counting system, and occasionally still Roman numerals and some types of tallies ([15], and see [51]). While their properties diverge, they also complement one another in ways that highlight different principles. This is why partial misalignment, for instance between number words and finger representation, may not just hinder but also help children to grasp key aspects when learning these systems [52]. Inter alia, finger counting marks 10 as a special number, while this is obfuscated in verbal systems, especially of the Germanic languages.

As reasons for the coexistence of entire systems are reviewed in detail elsewhere (notably [6,9,30,50]), the discussion here focuses on the coexistence of bases. Given the impact that bases have on system structure as well as cognitive representation and processing, and in view of their roles as benchmarks, we need to explain why they came to be used in parallel, what types of challenges this poses, and which factors lead to them persisting, changing—or being invented.

### (a) Origins and challenges of base diversity

As pointed out earlier, the systems for both counting and measuring are conditional on a reference unit and a scale along which quantity of units can be assessed. Yet, scales can operate with more than one type of unit, including bases, and these can vary (within limits) throughout history, across languages and cultures, and with context.

To begin with, systems differ because they are implemented in a variety of coexisting representational formats (or modalities), which in turn contribute to shaping system properties and equip the systems themselves for distinct and complementary functions [15]. Still, most numeration systems gravitate towards 10 as their base (or sub-base if the base is 20) [53]. This may be motivated by the practical ubiquity of 10 fingers, even though far from all body-based representations actually make use of the fingers in this exact way [54,55]. Base-10 also conveys cognitive advantages for counting and related arithmetic operations owing to being medium-sized, thus balancing the trade-off between two types of cognitive load: one involved in representing numerical information (the higher the base the better), the other in processing it (the lower the better).

Much like systems for counting, those for measuring were often grounded in the human body [30]. Unlike those for counting, however, the cognitive tools for measuring were originally designed and recruited for a wide array of distinct functions, including application in separate ranges of measurement, and therefore do not need to be convertible or to constitute a single coherent system. Even those in which higher units do relate to one another in a base-like manner do not have to do so with the same base as the numeration system on which they operate. Because ratios and proportions are arguably more important in measuring than in counting [49,50], such systems favour bases that have multiple divisors, such as 12 or 60, or various powers of 2, which afford simple manipulations such as halving or doubling [6].

Even within the same cultural tradition, systems for counting and for measuring therefore, almost by default, imply a coexistence of diverging bases. This works well so long as each system is predominantly applied to its own domain; challenges arise only when used in combination, such as when trying to ‘do the maths’ with units of time. In addition, systems that are not linked to spoken language (notations, abaci or measurement systems) have travelled across cultural traditions [9]. The incidents portrayed in the Introduction were caused by examples of such ‘travelling’ systems that came to be used for the same purpose—and collided.

Having to handle systems with diverging bases generates challenges, both internally (e.g. for workflows in mass production) and externally (for communication and coordination across cultural traditions). Such challenges become more hefty with increasingly complexifying fields, from basic science and engineering to international trade, that require commensurability across systems and aim to maximize accuracy, efficiency and speed of operation and production.

### (b) Persistence, unification and innovation

Whether or not these challenges lead to unification depends on several factors: how taxing cognitive load or switching habits is [40], how deeply embedded in cultural customs [9], how expensive in economic terms [6]—and eventually how much political will and power is mustered [31]. Most of these factors favour persistence. After all, these nonconvertible units of measure were once implemented for good reasons: they were readily available, easy to manipulate, with a distinct fit and differential advantages for the tasks at hand [30]. Once established and perpetuated over generations, they become so deeply rooted and entrenched in daily routines that they are hard and costly to change. This is why metrication required powerful intervention [31]—and was still only successful within certain limits, as outlined above.

It is worth pointing out that even when trying to harmonize two systems with diverging bases, there is no *a priori* reason for choosing one base over the other. In fact, during the push for metrication, several scientists and businessmen advocated adjusting the numeration system to measurement demands, rather than the other way around, by changing its base to 8 [56], 12 [57] or 16 [58].

This is in essence what Mangarevans did. While they retained the regular decimal system for enumeration more generally, they designed a new type of system, better adjusted to the halving and doubling of quantities at stake during tribute-giving, redistribution and trading [21]. Here, the parallel usage of systems both with primary units of diverging magnitude and with diverging base structure may not have been problematic at all. As the coexisting systems served explicitly distinct purposes, convertibility was not called for. Moreover, as any operation performed with them was executed publicly and in a ritualistic manner, indifferent to speed or efficiency, errors were unlikely, and even if they did occur, they did not inflict problems that were nearly as serious as the incidents described in the Introduction.

And yet, as resourceful as the Mangarevan mixed systems were in the context that stimulated their invention, they were still only tools, likely designed to tackle the challenges involved in mental arithmetic. When this context changed with the introduction of notational systems, the tools were abandoned.

## 7. Conclusion

Human interest in quantification has left behind a plurality of tools, including counting systems implemented in various representational formats and customary measurement systems designed for a wide scope of dimensions and for separate ranges within each of them. Embedded in this plurality was a juxtaposition of multiple bases and their structural, cognitive and practical ramifications. When bases and counting units with diverging numerical values clash, the consequences may be merely embarrassing, but may also be expensive and perilous. And yet, as parallel systems were designed to address practical needs, many of the same factors that gave rise to their plurality also work in favour of their persistence. Skilled users can become versed in productively combining them, as was described for the Greek mathematician, geographer and astronomer Ptolemaios (second century CE), who merged the representation of fractions in the Babylonian base-60 system with the representation of whole numbers in the Greek base-10 system, and particularly so when it was necessary to be precise [59]^3^.

In some cases, recurring tasks might even trigger innovations that add systems to the repertoire. One of the most impressive examples arose in a small-scale traditional context, in precolonial Mangareva. In order to tackle the challenges involved in measuring and counting large quantities in the absence of notation, Mangarevans designed mixed systems by combining decimal with binary steps that facilitated operations involved in tribute-giving and redistribution. In so doing, they manipulated the structure of their inherited numeration system to create a solid base for scaling up both the system at hand and what it would afford.

Numeration systems are cognitive tools that emerged to solve practical tasks. To understand why different tools—systems operating on diverging bases—have been used in parallel for the same task, we must not only achieve a better grasp of the concepts underlying counting and measurement, but also need to appreciate their cognitive implications, cultural significance and practical consequences. This endeavour can call our attention to the challenges lurking in system and base plurality and may help to keep ensuing mistakes and disasters at bay. It also provides us with insights into the contexts in which customary systems thrive, and where parallel usage of bases might do the trick. Cultural evolution is often conceived of as generating design without a designer, fine-tuning tools, as it were, in passing. In demonstrating how productive combining bases can be, rather than pitting them against each other, Mangarevans remind us that sometimes users take evolution into their own hands.

## Data Availability

This article has no additional data.

## Footnotes

^1^
By no means do all systems of quantification have a base-like structure (e.g. tally systems for counting or the systems for measuring temperature in either Celsius or Fahrenheit, as explained in §3a). These are acknowledged and considered of interest generally, but not included here. All claims made in the remainder of this paper apply to systems with base-like structures, unless specified otherwise.

^2^
These are perhaps better known as ‘Arabic digits’ or ‘Hindu-Arabic numerals’, but for the reasons laid out by Chrisomalis ([9, p. xv–xvii]), I follow him in denoting them ‘Western numerals’ in this paper.

^3^
My thanks to Reviewer 2 for alerting me to this wonderful example.

## Dedication

This article is dedicated to the late Martin G. Silverman and the late Sieghard Beller.
